# Prophylactic ceftizoxime for elective cesarean delivery at Soba Hospital, Sudan

**DOI:** 10.1186/1756-0500-6-57

**Published:** 2013-02-08

**Authors:** Bashier Osman, Amna Abbas, Mohamed A Ahmed, Magid S Abubaker, Ishag Adam

**Affiliations:** 1 Department of Pharmacology, Faculty of Pharmacy, University of Khartoum, Khartoum, Sudan; 2 Department of Obstetrics and Gynecology, Faculty of Medicine, University of Khartoum, P.O. Box 102, Khartoum, Sudan

**Keywords:** Prophylactic antibiotics, Ceftizoxime, Cesarean delivery, Sudan

## Abstract

**Background:**

A prophylactic antibiotic is recommended to reduce infection-related complication following cesarean delivery. There is a current debate on the time of prophylactic antibiotic in cesarean delivery.

**Methods:**

An opened randomized, controlled clinical trial was conducted at Soba hospital, Sudan to investigate the timing (pre-incision or after clamping of the umbilical cord) of ceftizoxime for elective cesarean delivery. The outcome measures were; the incidence of post-cesarean febrile and infection-related morbidity and neonatal outcomes between the two groups.

**Results:**

Hundred –eighty women (90 women in each arm of the study) received intravenous injection of 1 g of ceftizoxime as single dose either at pre-incision or after clamping of the umbilical cord. None of the women in either group had endometritis. One woman in the pre-incision group had chest infection. There was no significant difference in the incidence of wound infection between the two groups, 8 (6.7%) vs. 3 (3.3%); *P* = 0.2. Two babies in the pre-incision group (P = 0.497) had a low Apgar score (< 8) at 1 min. Similar number of neonate (15 in each arm) was admitted to nursery. There were no significant difference in the neonatal jaundice between the two groups, 5 (5.5%) vs. 4 (4.4%), *P* = 0.2. There was no perinatal death.

**Conclusions:**

There was no difference in the two regimens (pre-incision or post-clamping of the umbilical cord) of ceftizoxime as prophylactic for elective cesarean delivery.

**Trial registration:**

NCT01347593

## Background

There is an increase in the incidence of cesarean delivery and it is the most commonly performed major surgical procedure [1]. Cesarean delivery is the most important factor associated with postpartum infection, and carries a 5 to 20-fold increased risk of infection compared with vaginal delivery [2,3]. Wound infection is the most common infection-related complication following cesarean delivery which can be reduced by the use of prophylactic antibiotics [4].

Prophylactic antibiotics in women undergoing cesarean delivery substantially reduced the incidence of febrile morbidity [5]. Generally prophylactic antibiotics reduce surgical site infections and it is recommended to be administrated *prior* to surgical incision [6]. An exception to this pre-incision prophylactic approach is cesarean delivery. Because of concerns about the sequelae of fetal antibiotic exposure with pre-incision administration; the standard to prevent post-cesarean infection has been the administration of antibiotic prophylaxis after delivery of the baby and clamping of the umbilical cord [7]. Yet, recent observations have challenged this policy of giving antibiotic prophylaxis after delivery of the baby and clamping of the umbilical cord [8]. Thus, there is a current debate on the time of prophylactic antibiotic in cesarean delivery. Investigating the time of prophylactic antibiotic in cesarean delivery is highly needed before changing the policy from post-clamping administration of prophylactic antibiotics to pre-incision administration.

A first generation cephalosporin (cefazolin) has been recommended as the regimen of choice for prophylactic antibiotic in cesarean delivery because of increasing microbial resistance [9] However, 2nd or 3rd generation cephalosporins have been evaluated as prophylactic antibiotic in cesarean delivery with encouraging results [10]. Ceftizoxime (a 3rd generation cephalosporin) has shown an excellent profile against surgical infecting organisms [11]. The current study was conducted to investigate the timing of prophylactic ceftizoxime for elective cesarean delivery at Soba Hospital, Sudan and so as to add to the previous reaches on prophylactic antibiotics for elective cesarean delivery in Sudan [12].

## Methods

A mono-centric opened randomized, controlled clinical trial was carried- out during the period May –August 2011 at Soba Hospital.

### Patients selection

Patients who were planned for elective cesarean delivery for various reasons e.g. repeated scars, breech and low lying placenta were enrolled in the study. Women were excluded from the study if they had severe anaemia, twins, diabetes mellitus, impaired glucose test, received antibiotics within two weeks prior to the operation; if they had any visible infection at any site or elevated temperature at the time of the operation; if they were allergic to drug used; or if they were not wish to participate in the study.

### Treatment arms

Women were randomized by computer generated block-randomization and concealed envelope system was used to allocate the patients to receive intravenous injection of 1 g of ceftizoxime as single dose either at the interval of 40 minutes pre-incision or post -clamping the umbilical cord. After giving consent, a complete history of the participants was taken using a standard questionnaire. A physical examination was performed.

### Follow-up measurement

The primary outcome measure was the incidence of post-operative febrile morbidity, defined as an oral temperature of ≥ 38.0 °C on two occasions at least four hours apart, excluding the first 24 hours; it can be due to post-operative infection, which includes: endometritis (fever, uterine tenderness and abnormal lochia), wound infection (fever, cellulitis and exudates), pelvic abscess, peritonitis (elevated temperature, tachycardia, abdominal distension and pain with guarding and rigidity aggravated by moving and breathing with absent bowel sounds at the onset of paralytic ileus) other febrile morbidity, e.g. urinary tract infection, chest infection, malaria [13].

Secondary outcome measures were the neonatal outcomes: the incidence of low APGAR score (< 8) at 1 min, neonatal jaundice and admission to the nursery.

Once febrile morbidity was identified, women were examined thoroughly to localize the potential source of infection (tonsils, breasts, chest, abdomen and pelvis). Urine analysis (followed by urine culture and sensitivity testing if the result of examination was suggestive of infection); total white blood cell count. Blood and cervical swabs were sent for culture (MacConkey Agar) and sensitivity testing. Blood films (thin and thick) were taken by finger pricks and Giemsa stained to confirm or to exclude malaria. The policy in the hospital is to treat post-caesarean delivery febrile morbidity (endometritis, peritonitis and pelvic abscess) with amoxicillin + clavulanic acid and metronidazole 500 mg 8 hourly for 7 days. If there was no response, these drugs were changed to antibiotics in accordance with the result of the culture sensitivity test. If no complications, women were discharged from the hospital on the fourth day and were instructed to presented at any time if there is any complication, otherwise they were seen one, two and 6 weeks later in the post delivery clinic where they were examined for wound infection. The follow-up was performed by a trained medical officer who was blinded to the allocation to pre-incision or post-cord clamping (to avoid bias).

### Ethics

The study received ethical clearance from the Committee of Research Board of Soba Hospital, Sudan.

### Statistical analyses

Data were entered in the computer using SPSS for windows version16.0 and double checked before analysis. Means and proportions of the socio-demographic and obstetrical characteristics were calculated and compared between the two groups using student *t* and *X*^2^ tests, respectively. A probability value of < 0.05 was considered as statistically significant.

## Results

One hundred-eighty (90 women in each arm) women were enrolled to the study for various indication of cesarean delivery (Table 1). The two groups were well- matched in the age, parity, weight, height, gestational age and temperature (Table 2).

**Table 1:** Indications of cesarean delivery in the two groups*

**Indication of cesarean delivery**	**Women who received pre-incision ceftizoxime (n =90)**	**Women who received ceftizoxime after clamping the umbilical cord (n =90)**	**P**
Repeated cesarean delivery	55 (61.1)	61 (67.8)	0.1
Breech presentation	10 (11.1)	8 (8.9)	0.6
Hypertensive disorder	2 (2.2)	1 (1.1)	0.5
Bad obstetrics events	7 (7.8)	11 (12.2)	0.3
Others	16 (17.8)	9 (10)	0.1

*data were shown as n (%).

**Table 2:** Mean (SD) of the basic characteristics of women who received ceftizoxime as prophylaxis for elective cesarean section

**Variables**	**Women who received pre-incision ceftizoxime (n =90)**	**Women who received ceftizoxime after clamping the umbilical cord (n =90)**	**P**
Age, years	30.5 (7.4)	32.2 (5.2)	0.08
Gravidity	3.5 (1.6)	3.9 (2.1)	0.1
Parity	1.9 (1.5)	2.1 (1.5)	0.4
Weight, kg	77.4 (13.7)	79.4 (13.7)	0.3
Height, cm	158.3 (5.3)	159.6 (6.0)	0.1
Gestational age, weeks	38.2 (1.1)	38.3 (0.9)	0.7
Temperature, °C	36.9 (0.5)	36.9 (0.4)	0.5

There is no significant difference in the maternal and perinatal outcomes measures, (Table 3). While none of the women who received ceftizoxime of after clamping of the umbilical cord had post-operative febrile morbidity, one woman who received the drug pre-incision had post-operative febrile morbidity which was due to chest infection (*P* = 0.3). None of the women in both groups had endometritis. There was no significant difference in the incidence of wound infection between the two groups, 8 (6.7%) vs. 3 (3.3%); *P* = 0.2. All of these wound infections observed during the follow-up after the patients’ discharge from the hospital. While none of the babies in the received ceftizoxime of after clamping of the umbilical cord had low Apgar score (< 8) at 1 min, two babies in the pre-incision group (*P* = 0.497) had low Apgar score (<8) at 1 min. A Similar number of neonate (15 in each arm of the study, *P* = 0.9) was admitted to nursery (for the mean [SD] of 1.9 [0.7] vs. 1.7 [0.8]days; p = 0.742 in the two groups, respectively) due to jaundice, low Apgar score, low birth weight and for routine check-up for those babies born to mothers with had bad obstetric events. There were no significant difference in the neonatal jaundice between the two groups, 5 (5.5%) vs. 4(4.4%), *P* = 0.2.

**Table 3:** Number (%) of maternal and perinatal outcome in women who received ceftizoxime as prophylaxis for elective cesarean section

**Variables**	**Women who received ceftizoxime pre-incision (n =90)**	**Women who received ceftizoxime after clamping the cord (n =90)**	**P**
Post-operative febrile morbidity	1(1.1)	0 (0)	1.0
Superficial wound infection	8 (6.7)	3 (3.3)	0.2
Skin rash	0 (0)	1 (1.1)	1.0
Jaundice	5 (5.5)	4 (4.4)	1.0
Admission to the nursery	15 (16.7)	15 (16.7)	0.1

## Discussion

The main finding of the current study was; no significant difference between the incidence of post-operative febrile morbidity, superficial wound infections, neonatal jaundice and neonatal admission to the nursery between the two groups of women who received ceftizoxime either pre-incision or after clamping of the umbilical cord. It has been advised that prophylactic antibiotics should be given before surgical incision to prevent post-operative surgical infections [6]; an exception in this generalization is the cesarean delivery, where the recommendation is to use prophylactic antibiotics after clamping of the umbilical cord. A recent survey that described practice of antibiotic prophylaxis for cesarean delivery among American maternal-fetal medicine physicians showed that; preoperative administration of antibiotic prophylaxis was the commonest practice which was reported by 84.6% of the physician compared to 15.0% who reported giving antibiotic prophylaxis after umbilical cord clamping [14].

The current debate of antibiotics commonly used for cesarean prophylaxis is the concern that antibiotics rapidly transferred to the newborn and the fetal exposure to antibiotics might mask infection in the neonate and the possibility of the selection of resistant organisms [9]. Thus, sepsis work-ups on neonates who were exposed to antibiotics immediately prior to the incision have been practiced [15]. Furthermore, previous old studies of antibiotic prophylaxis for cesarean delivery suggested that, while pre-incision administration did not reduce post-cesarean infection, it did increase invasive neonatal sepsis evaluations and costs [15,16]. However, recently Kaimal et al., [17], in a cohort study of large number of caesarean delivery (1316) reported that a policy change in timing of antibiotic prophylaxis from post-clamping to pre-incision resulted in a reduction of 60%, 50% and 80% in the rate of surgical site infection, endometritis and cellulitis, respectively. Another recent study on a change in policy from post-clamping administration of prophylactic antibiotics to pre-incision administration showed no difference incidence of endometritis (3.9% vs. 2.2%), and wound infection (3.6% vs. 2.5%) in the two groups of women who received cefazolin post-clamping antibiotics vs. pre-incision [18]. Moreover, recent study documented that, the use of pre-incision antibiotics was not associated with an increase in neonatal sepsis, sepsis work-up, and admission or length of stay in the Neonatal Intensive Care Unit [19].

In the current study, there were 6.7% vs. 3.3%, P = 0.2 wound infection in the group of women who received ceftizoxime post-clamping antibiotics vs. pre-incision. The diagnose of these wound infections during the follow-up period after the patients discharged might indicate the true incidence of these events. Previous study showed that, up to 80% of infections occur after discharge from the hospital [20-22]. Thus, post- cesarean deliveries infection rates may be underestimated if based on hospital discharge records. The prevalence of wound infection varies according to the setting itself and it was reported among10% of cesarean deliveries despite recommended antibiotic prophylaxis [7,20,21].

In the current study 15 babies in each arm of the study were admitted to the nursery. Perhaps, these rates might has been influenced by the decision of the operation itself where 19 of these cesarean deliveries were performed for bad obstetrical events i.e. on the baby’s behalf.

### Study limitations

One limitation of this study is the sample size was not powered to show either equivalence or superiority as the sample size was not calculated to address these; perhaps larger size study (around 4000 patients) is needed to according to open Epio-epidemioligic calculator [23], with the proportion of those women with post-operative febrile morbidity (2 vs. 3%) within 3–5 percentage points of the true proportion and 80% power. The other limitations of this study were; cost was not investigated and there was no other type of antibiotic used as control.

## Conclusion

Both regimens of pre-incision or post-clamping of the umbilical cord of ceftizoxime were effective prophylactic for elective cesarean delivery.

## Competing interest

Authors have competing interest.

## Authors’ contributions

BO and IA designed the study. AA and MAA conducted the clinical work. MSA conducted the statistical analyses. All authors contributed to and approved the final manuscript.
